# Top motor and non-motor complaints in patients with Parkinson's disease

**DOI:** 10.3389/fnagi.2025.1664934

**Published:** 2025-10-22

**Authors:** José Fidel Baizabal-Carvallo, Marlene Alonso-Juarez, Robert Fekete

**Affiliations:** ^1^Department of Sciences and Engineering, University of Guanajuato, León, Mexico; ^2^Instituto Politécnico Nacional, Mexico City, Mexico; ^3^New York Medical College, Valhalla, NY, United States

**Keywords:** Parkinson's disease, levodopa, tremor, symptoms, signs

## Abstract

**Background:**

Parkinson's disease (PD) is characterized by several motor and non-motor manifestations. These variable neurological complaints have diverse intensity and cause various degrees of disability.

**Objective methods:**

We aimed to assess the top three most troublesome neurological complaints in patients with PD and correlated them with demographic and clinical variables, including the cognitive status assessed by the Montreal Cognitive Assessment (MoCA). Patients were asked about their three most troublesome neurological complaints after reviewing all motor and non-motor symptoms. Responses were divided into three tiers.

**Results:**

We studied 230 consecutive patients with PD. There were 130 (56.5%) male patients, with the mean age at evaluation of 67.7 ± 11.06 years. The most common neurological complaints in the top tier were (1) tremor (*n* = 80, 34.8%), (2) gait problems (*n* = 37, 16.1%), and (3) dyskinesia (*n* = 17, 7.4%). Speech difficulties, dyskinesia, and insomnia became more prominent 5 or 10 years after disease onset. A total of 159 (69.1%) patients reported “at least one non-motor symptom” among their top-3 tier complaints. Pain (*n* = 29, 12.6%), anxiety (*n* = 25, 10.8%), and insomnia (*n* = 25, 10.8%) were the most common non-motor symptoms. The presence of non-motor symptoms in the top three tiers was associated with decreased cognitive status (MoCA <25 points), but not with age, sex, or disease evolution time. Cognitive impairment was a significant predictor of non-motor symptoms in their top three tiers [odds ratio [OR]: 3.88 (95% CI: 1.647 to 9.169)]. Cluster analysis of patients with at least one non-motor symptom identified four groups: male or female patients with short evolution time, a postural-instability gait difficulty (PIGD) phenotype, and a dyskinetic group. The latter two were associated with a higher frequency of fatigue, insomnia, pain, and anxiety.

**Conclusion:**

Overall, tremor was the most troublesome symptom in patients with PD, though high variability was observed. Approximately 69% of patients had at least one non-motor symptom among their top-3 complaints, which was associated with abnormal cognitive status. Speech difficulties, dyskinesia, and insomnia became more prominent with disease progression. Among patients with non-motor symptoms in the top three tiers, those with a PIGD phenotype or prominent dyskinesia exhibited a higher frequency of fatigue, insomnia, pain, and anxiety, suggesting a clustering effect of non-motor symptoms with these motor presentations.

## Introduction

Parkinson's disease (PD) is a neurological degenerative disorder characterized by the loss of dopaminergic neurons following the accumulation of α-synuclein in the form of Lewy bodies. Although motor manifestations such as rigidity, bradykinesia, and tremor are paramount for the diagnosis, several other motor and non-motor manifestations commonly affect these patients (Jankovic, 2008). These diverse clinical manifestations can vary in their level of impact on activities of daily living, social functioning, and overall quality of life, with significant variability among individuals with PD (Staunton et al., 2022). In our experience, patients with PD are usually able to provide a hierarchical account of these manifestations based on how bothersome they perceive them to be.

However, there is limited information on which clinical manifestations of PD are perceived as the most troublesome or disabling by patients. In this study, we aimed to assess the top three clinical neurological and psychiatric complaints reported by consecutive patients with PD presenting for evaluation at a Movement Disorders Clinic. Additionally, we explored the associations between these complaints with sex, age at onset and at evaluation, evolution time, severity of motor manifestations, stage of the disease, antiparkinsonian medication status, and cognitive impairment.

## Materials and methods

We made a retrospective review of patients with PD attending a tertiary-care center for movement disorders. The inclusion criteria were patients of any age and both sexes diagnosed with PD according to the Queen Square Brain Bank (QSBB) criteria (Hughes et al., 1992). The exclusion criteria were patients without a clear diagnosis of PD or patients with parkinsonism secondary to another disorder (i.e., atypical parkinsonism).

Demographic variables were recorded in each case, including sex and age at the first evaluation. We considered the onset of PD when the first motor manifestation was perceived by the patient or a family member. The evolution time was calculated from the first motor manifestation. We determined the three most troublesome manifestations related to PD in each patient. To achieve this goal, we first systematically reviewed the presence of all motor symptoms in PD, including motor complications such as dyskinesia and motor fluctuations. Non-motor manifestations were also systematically reviewed (Poewe, 2008), including: (1) cognitive and neuropsychiatric complaints (e.g., anxiety and depression), (2) sleep problems such as insomnia and REM sleep behavior disorder (RBD); (3) gastrointestinal symptoms, including nausea, vomiting, and constipation; (4) sensory manifestations, including pain; (5) autonomic dysfunctions, such as prominent diaphoresis and orthostatic hypotension; and (6) sexual dysfunction (Poewe, 2008). Patients were also encouraged to name any other neurological manifestation among their top-3 tier complaints. The neurological manifestations reviewed in each case are summarized in Supplementary material. After reviewing all these clinical manifestations, patients were encouraged to spontaneously classify their most bothersome or troublesome symptoms. This methodology aimed to reduce the risk of recall bias across all neurological symptoms. In case a particular patient was able to name only one or two symptoms, these were recorded as top-tier or second-tier responses, with no third response recorded. As patients may use diverse expressions to refer to the same manifestations, they were homogenized using unifying terminology for statistical analysis and scientific communication (Supplementary material).

The evaluation of parkinsonism was performed by a movement disorders specialist (JFB-C) with the Movement Disorders Society Unified Parkinson's Disease Rating Scale part III or motor score (MDS-UPDRS-III) (Goetz et al., 2008). Patients were evaluated in the medication “off” state in case they were naive to dopaminergic drugs or before the next levodopa cycle in case they were already taking this medication. The disease stage by functional impairment was assessed with the modified Hoehn and Yahr (mHY) scale (Hoehn and Yahr, 1967). The cognitive evaluation was carried out with the Montreal Cognitive Assessment (MoCA) at the time of evaluation (range: 0–30 points), where a lower score indicates a worse cognitive impairment (Nasreddine et al., 2005). For this study, a score of ≥25 points was considered normal (Milani et al., 2018). Antiparkinsonian medications were registered in each case at the time of enrollment. Antiparkinsonian-naïve status was also considered when a patient had never taken any dopaminergic medication (including levodopa), amantadine, anticholinergic, or a monoamine oxidase inhibitor. The levodopa equivalent daily dose (LEDD) was determined for patients taking antiparkinsonian medications at the time of enrollment, according to published guidelines (Schade et al., 2020). The study was approved by the Internal Review Board of Santé Medical Tower, and patients provided written informed consent to participate in the study.

## Statistics

Data were summarized as percentages, means with standard deviations, or medians with interquartile ranges, depending on the normality of the distribution. The Kolmogorov–Smirnov test was used to assess the normal distribution of continuous variables. The independent *t*-test or the non-parametric Mann–Whitney *U*-test was used to compare continuous variables between groups. The *X*^2^ test with Yates's continuity correction or Fisher's exact test was used, when appropriate, to compare nominal or ordinal data between groups. Data were evaluated after stratifying by sex (male and female), time of evolution ( ≤ 5 years or >5 years and ≤ 10 or >10 years), and impaired cognitive status (MoCA < 25 points and ≥25 points).

Additionally, we performed a two-step cluster analysis for the group of patients with at least one non-motor symptom in the top three tiers. The Bayesian Schwarz method was used as a grouping criterion. Variables showing the main indicators of PD severity, including axial symptoms and dyskinesia, were selected for grouping purposes (*n* = 9, variables). An analysis of variance (ANOVA) was used to describe differences between groups. All statistical evaluations were performed using SPSS version 22. A *P*-value of < 0.05 was considered significant (^*^) and a *P*-value of < 0.01 was considered highly significant (^**^).

## Results

**Figure 1:**
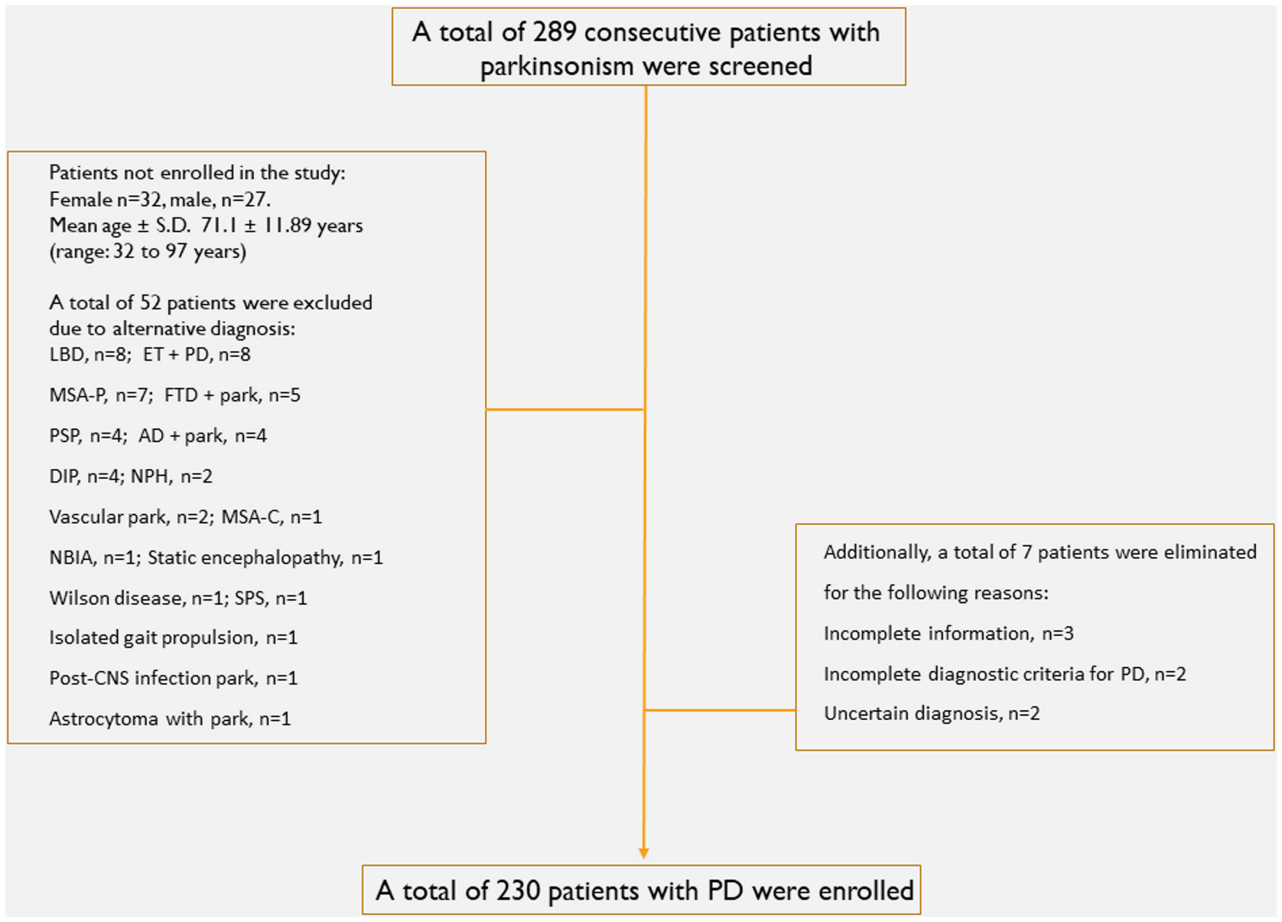
Cohort attrition of patients enrolled in the study. AD, Alzheimer disease; DIP, drug-induced parkinsonism; ET, essential tremor; FTD, frontotemporal dementia; LBD, Lewy body dementia; MSA (P/C), multiple system atrophy (parkinsonian type/cerebellar type); NBIA, neurodegeneration with brain iron accumulation; NPH, normal pressure hydrocephalus; Park, parkinsonism; PD, Parkinson's disease; PSP, progressive supranuclear palsy; SPS, stiff-person syndrome.

We evaluated 289 consecutive patients with parkinsonism. Fifty-two patients were excluded from the study because they had diagnoses other than PD. Additionally, seven patients were excluded due to incomplete information, parkinsonism that did not meet the diagnostic criteria for PD, or an uncertain diagnosis (Figure 1). A total of 230 patients were enrolled in the study. There were 130 (56.5%) male patients and 100 (43.5%) female patients, with the mean age at evaluation of 67.7 ± 11.06 (range: 33 to 90 years) and the mean evolution time of 6.61 ± 6.58 years (range: 2 months to 45 years).

A response for the first tier of the most troublesome symptom was registered in 230 (100%) patients; a response for the second tier symptom was registered in 229 (99.56%); whereas a response for the third tier symptom was registered in 207 (90%) patients. There were 29 different symptoms in the first tier, 34 different symptoms in the second tier, and 35 different symptoms in the third tier.

**Figure 2:**
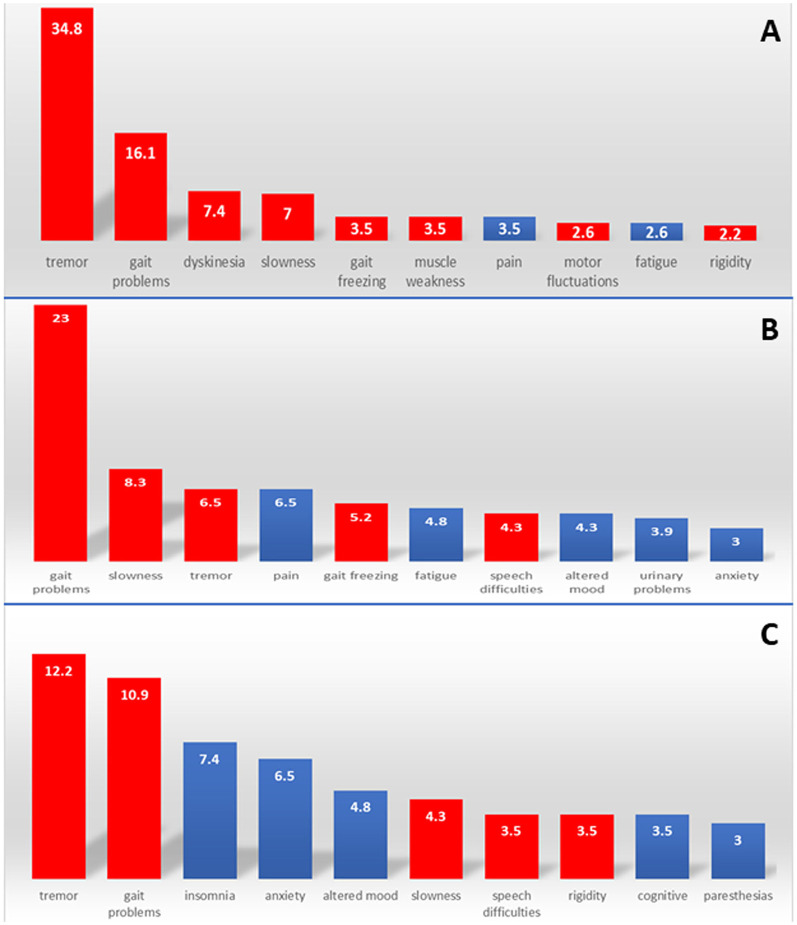
Top-10 neurological complaints in the top three tiers. Red: motor complaints, blue: non-motor complaints. **(A)** First tier; **(B)** second tier; **(C)** third tier.

**Figure 3:**
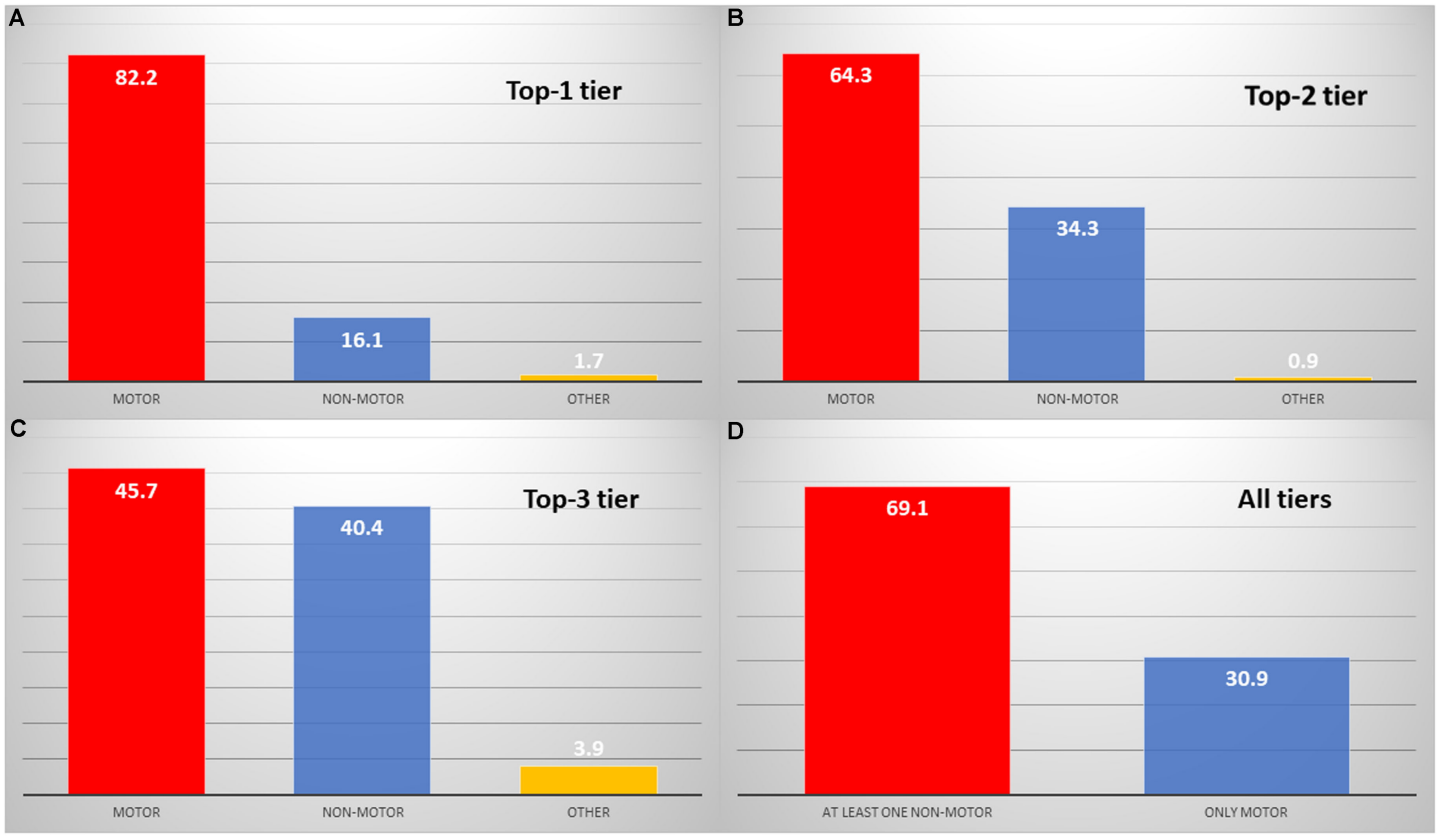
Motor vs. non-motor complaints in the top one tier: **(A)** top two tiers: **(B)** top three tiers: **(C)** and overall: **(D)** Red: motor complaints, blue: non-motor complaints, yellow: other complaints not related to Parkinson's disease.

When considering the first tier, the most common neurological complaints were (1) tremor (*n* = 80, 34.8%), followed by (2) gait problems (*n* = 37, 16.1%) and (3) dyskinesia (*n* = 17, 7.4%) (Supplementary Tables 1–3). Overall, the frequency of symptoms was more variable for the second and third tiers (Figure 2). The frequency of non-motor symptoms increased from *n* = 37 (16.1%) in the first tier to *n* = 79 (34.3%) in the second tier and *n* = 93 (40.4%) in the third tier (Figure 3). Tremor, gait problems, and slowness were common top complaints at any time during the disease course. Speech difficulties, dyskinesia, and insomnia became prominent after 5 or 10 years of the disease onset (Table 1).

**Table 1:** Distribution of the most common complaints according to the evolution time of Parkinson's disease.

	** < 5-year evolution**	**≥5-year evolution**	*P*-value^*^	** < 10-year evolution**	**≥10-year evolution**	*P*-value^*^
First-tier symptoms	Tremor *n* = 58	Tremor *n* = 22	< 0.001^**^	Tremor *n* = 69	Dyskinesia *n* = 13	0.005^**^
	Gait problems *n* = 22	Dyskinesia *n* = 15		Gait problems *n* = 29	Tremor *n* = 11	
	Slowness *n* = 12	Gait problems *n* = 15		Slowness *n* = 13	Gait problems *n* = 8	
	Total responses: 131	Total responses: 99		Total responses: 173	Total responses: 57	
Second-tier symptoms	Gait problems *n* = 33	Gait problems *n* = 20	0.035^*^	Gait problems *n* = 39	Gait problems *n* = 14	0.173
	Pain *n* = 13	Slowness *n* = 9		Slowness *n* = 15	Slowness *n* = 4	
	Slowness *n* = 10	Speech difficulties *n* = 8		Pain *n* = 14	Speech difficulties *n* = 4	
	Total responses: 130	Total responses: 99		Total responses: 172	Total responses: 57	
Third-tier symptoms	Tremor *n* = 15	Tremor *n* = 58	0.166	Gait problems *n* = 20	Tremor *n* = 9	0.739
	Gait problems *n* = 12	Gait problems *n* = 22		Tremor *n* = 19	Insomnia *n* = 7,	
	Anxiety *n* = 10	Insomnia *n* = 11		Anxiety *n* = 11	Gait problems *n* = 5	
	Total responses: 118	Total responses: 89		Total responses: 157	Total responses: 50	

^*^P-values are for comparisons of symptom distribution: ^*^significant: *P* < 0.05, ^**^highly significant: *P* < 0.01.

Overall, 159 (69.1%) patients endorsed “at least one non-motor symptom” in their top 3 tiers of most troublesome symptoms, with *n* = 99 (43.0%) patients having one non-motor symptom, *n* = 56 (24.3%) having two non-motor symptoms, and *n* = 4 (1.7%) having three non-motor symptoms in their top three tiers. Pain was the most common non-motor symptom in the top three tiers, *n* = 29 (12.6%), followed by anxiety, *n* = 25 (10.8%), insomnia, *n* = 25 (10.8%), altered mood, *n* = 22 (9.6%), and fatigue, *n* = 22 (9.6%) (Supplementary Table 2).

When comparing patients with “at least one non-motor symptom” in their top three tiers vs. patients with only motor symptoms in their top 3 tiers, no differences were observed in sex distribution, evolution time, presence of dyskinesia, individual motor subscores in the MDS-UPDRS-III, mHY stage, antiparkinsonian medication-naïve, and LEDD (Table 2). Patients with only motor symptoms in their top three tiers had a statistically significant trend toward younger age (*P* = 0.057) and higher MDS-UPDRS-III total scores (*P* = 0.091). However, patients with “at least one non-motor symptom” had lower median MoCA scores: 22 vs. 25 (*P* = 0.002), with a greater proportion of patients having an abnormal MoCA (< 25) score: 67% vs. 34.4% (*P* = 0.001) (Table 2).

**Table 2:** Stratification by sex, evolution time, and cognitive status of patients with at least one non-motor symptom.

	**At least one non-motor: Yes, *n* = 159 (%)**	**At least one non-motor: No, *n* = 71 (%)**	***P*-value**
Age	70.0 (62.0, 75.0)	64.0 (57.0, 76.0)	0.057
Male patients	89 (56)	41 (57.7)	0.802
Evolution time^*^ **(years)**	4.0 (2.0, 9.0)	5.0 (2.0, 10.5)	0.621
≥5 years of evolution	67 (42.2)	32 (45)	0.678
≥10 years of evolution	37 (23.3)	20 (28.2)	0.427
MoCA abnormal (< 25)	106 (67)	24 (34.4)	0.001^**^
MoCA^*^ score	22.0 (17.0, 25.0)	25.0 (23.5, 27.00)	0.002^**^
**MDS-UPDRS-III** ^*^			
Language	1.0 (0.0, 1.0)	1.0 (0.0, 2.0)	0.510
Facial expression	1.0 (0.0, 2.0)	1.0 (0.0, 2.0)	0.539
Rigidity (0–20)	7.0 (4.0, 10.0)	8.0 (4.0, 12.0)	0.125
Bradykinesia (0–40)	12.0(6.0, 18.0)	13.5 (7.0, 22.0)	0.174
Rising from chair	1.0 (0.0, 2.0)	1.0 (0.0, 2.0)	0.367
Gait	1.0 (1.0, 2.0)	2.0 (1.0, 3.0)	0.184
Freezing	1.0 (0.0, 1.0)	0.0 (0.0, 1.0)	0.148
Postural stability	2.0 (1.0, 2.0)	2.0 (1.0, 2.0)	0.714
Posture	1.0 (0.0, 2.0)	1.0 (1.0, 2.0)	0.353
Postural tremor (0–8)	1.0 (0.0, 2.0)	1.0 (0.0, 2.0)	0.222
Action tremor (0–8)	0.0 (0.0, 2.0)	1.0 (0.0, 2.0)	0.133
Rest tremor (0–20)	2.0 (0.0, 3.5)	2.0 (0.0, 4.0)	0.215
Tremor persistence (0–4)	1.0 (0.0, 3.0)	2.0 (0.0, 3.0)	0.591
Hypokinesia	1.0 (0.0, 2.0)	1.0 (1.0, 2.0)	0.433
Total score	35.18 ± 18.83	41.01 ± 22.15	0.091
Hoehn-Yahr^*^	3.0 (3.0, 4.0)	3.0 (3.0, 4.0)	0.344
Presence of dyskinesia	24 (15)	9 (12.7)	0.780
Antiparkinsonian naïve	47 (29.6)	24 (33.8)	0.520
**Medications**			
Levodopa	100 (62.9)	40 (56.3)	0.347
MAOIs	21 (13.2)	1 (1.4)	0.010^*^
Amantadine	14 (8.8)	4 (5.6)	0.574
Dopaminergic agonists	42 (26.4)	21 (29.6)	0.619
Anticholinergics	10 (6.3)	6 (8.4)	0.580
LEDD	590.1 ± 604.9	537.7 ± 600.6	0.544

LEDD, levodopa equivalent daily dose; MAOI, monoamine oxidase inhibitors; MoCA, Montreal Cognitive Assessment. ^*^Significant: *P* < 0.05, ^**^highly significant: *P* < 0.01.

When considering the top three tiers, no differences between male and female patients were observed for “at least one non-motor symptom” in the first (*P* = 0.526) and third tiers (*P* = 0.423), but a trend for a higher frequency of female vs. male patients (42.4% vs. 30%) was observed in the second tier (*P* = 0.051) (Table 3). No differences were observed in evolution time for any tier, whereas abnormal MoCA was more common in patients with at least one non-motor symptom in the first (*P* = 0.017) and second tiers (*P* = 0.025) (Table 3). A decreased cognitive status (MoCA < 25 points) was a predictor of patients complaining of at least one non-motor symptom in their top three tiers, OR: 3.88 (95% C.I. 1.647 to 9.169).

**Table 3:** Stratification by sex, evolution time, and cognitive status of patients within tiers.

	**Motor symptom: patients, *n* (%)**	**Non-motor symptom: patients, *n* (%)**	**Totals patients, *n***	***X^2^ value***	***P*-value**
**First tier**					
Male patients	105 (80.8)	25 (19.2)	130	0.403	0.526
Female patients	84 (84)	16 (16)	100		
**Second tier**					
Male patients	91 (70)	39 (30)	130	3.795	0.051
Female patients	57 (57.6)	42 (42.4)	99		
**Third tier**					
Male patients	57 (48.3)	61 (51.7)	118	0.643	0.423
Female patients	48 (53.9)	41 (46.1)	89		
**First tier**					
< 5 years of evolution	111 (84.7)	20 (15.3)	131	1.360	0.243
≥5 years of evolution	78 (78.8)	21 (21.2)	99		
**Second tier**					
< 5 years of evolution	79 (60.8)	51 (39.2)	130	1.960	0.162
≥5 years of evolution	69 (69.7)	30 (30.3)	99		
**Third tier**					
< 5 years of evolution	58 (49.2)	60 (50.8)	118	0.271	0.602
≥5 years of evolution	47 (52.8)	42 (47.2)	89		
**First tier**					
< 10 years of evolution	143 (82.4)	30 (17.3)	173	0.112	0.738
≥10 years of evolution	46 (80.7)	11 (19.3)	57		
**Second tier**					
< 10 years of evolution	107 (62.2)	65 (37.8)	172	1.770	0.183
≥10 years of evolution	41 (71.9)	16 (28.1)	57		
**Third tier**					
< 10 years of evolution	80 (51)	77 (49)	157	0.014	0.906
≥10 years of evolution	25 (50)	25 (50)	50		
**First tier**					
MoCA ≥25	90 (88.2)	12 (11.8)	102	8.150	0.017^*^
MoCA < 25	99 (77.3)	29 (22.7)	128		
**Second tier**					
MoCA ≥25	72 (72.7)	27 (27.3)	99	7.387	0.025^*^
MoCA < 25	76 (58.5)	54 (41.5)	130		
**Third tier**					
MoCA ≥25	50 (54.9)	41 (45.1)	91	0.882	0.643
MoCA < 25	55 (47.4)	61 (52.6)	116		

MoCA, Montreal Cognitive Assessment. ^*^Significant: *P* < 0.05, ^**^highly significant: *P* < 0.01.

A data-driven cluster analysis of patients reporting at least one non-motor symptom identified four groups: (1) a male, short-evolution time group (*n* = 63); (2) a female, short-evolution time group (*n* = 44); (3) a postural-instability gait difficulties group (*n* = 29); and (4) a dyskinetic group (*n* = 22) (Table 4). Tremor and gait problems were the two most troublesome complaints in groups 1, 2, and 3, while pain and dyskinesia were most common in group 4. Gait problems were the top complaints in the second tier for groups 1, 2, and 3, but still were the most common in the third tier (Table 4).

**Table 4:** Cluster analysis of patients with at least one non-motor symptom in their top three tiers of most troublesome symptoms.

**Variables**	**Male patients, short evolution group, *n* = 63**	**Female patients, short evolution, group, *n* = 44**	**PIGD group, *n* = 29**	**Dyskinetic group, *n* = 22**	***X*^2^/F value**	*P-*value^*^
Sex	Male patients, 100%	Female patients, 100%	Female patients, 55,2%	Male patients, 59.1%	1.181	0.319
Evolution time	5.66 ± 5.28	5.07 ± 4.97	9.44 ± 4.06	8.82 ± 8.51	3.662	0.014^*^
Rigidity score	7.12 ± 2.69	3.87 ± 2.85	13.89 ± 3.55	10.03 ± 3.12	48.627	< 0.001^**^
Bradykinesia score	12.68 ± 4.11	4.54 ± 2.91	32.89 ± 4.91	22.52 ± 5.11	204.447	< 0.001^**^
Gait score	1.76 ± 0.88	0.69 ± 0.73	3.78 ± 0.67	2.45 ± 1.02	49.397	< 0.001^**^
Freezing score	0.49 ± 1.06	0.04 ± 0.28	2.89 ± 1.69	1.28 ± 1.62	22.693	< 0.001^**^
MDS-UPDRS-III total score	34.46 ± 5.53	16.96 ± 6.60	82.11 ± 12.98	54.86 ± 7.25	349.925	< 0.001^**^
Hoehn–Yahr score	3.16 ± 0.81	2.62 ± 0.70	4.44 ± 0.73	3.83 ± 0.71	25.091	< 0.001^**^
Dyskinesia	0	0	6.9%	100%	1.407	0.243
First tier, top complaints (%)	Tremor (38)	Tremor (45,5)	Tremor (20.7)	Pain (22.7)	112.458	0.006^**^
	Gait problems (14.3)	Gait problems (18.2)	Gait problems (20.7)	Dyskinesia (18.2)		
	Slowness (7.9)		Fatigue (10.3)	Tremor (13.6)		
Second tier, top complaints (%)	Gait problems (19)	Gait problems (18.2)	Gait problems (24.1)	Speech diff (13.6)	99.912	0.372
	Pain (11.1)	Urinary prob (11.4)	Slowness (10.3)	Anxiety (13.6)		
	Fatigue (11.1)	Pain (9.1)	Pain (10.3)	Tremor (9.1)		
Third tier, top complaints (%)	Anxiety (9.5)	Altered mood (18.2)	Gait problems (13.8)	Insomnia (18.2)	105.762	0.123
	Paresthesia (9.5)	Anxiety (13.6)	Speech (13.8)	Speech (9.1)		
	Insomnia (7.9)	Tremor (13.6)	Insomnia (10.3)	Hypotension (9.1)		

^*^P-value is only descriptive, as groups were generated by maximizing differences. ^*^Significant: *P* < 0.05, ^**^highly significant: *P* < 0.01.

## Discussion

In the present study, we aimed to identify what are the most troublesome neurological symptoms in a cohort of patients with PD. Responses varied between 29 and 34 different symptoms acknowledged in the top 3 tiers. Motor symptoms predominated over non-motor symptoms, with tremor, gait problems, and slowness being the most frequent complaints in the top 3 tiers, whereas pain was the fourth overall complaint and the most common “non-motor” symptom. At least one non-motor symptom in the top three tiers was mentioned by a high proportion (69.1%) of patients. While sex, age, evolution time of PD, antiparkinsonian medication status, and LEDD were not predictive of non-motor symptoms in the top 3 tiers, abnormal cognitive status, as measured by the MoCA, was a significant predictor of at least one top-tier non-motor symptom.

In one study involving 40 patients with PD assessed via online interviews, tremor, fine motor movements, and slowness were the most “bothersome” symptoms (Mammen et al., 2023). The symptom frequency was similar to our study, except for “fine motor movements.” Instead, gait problems were reported in our cohort. While these symptoms may have diverse impacts on ADL, quality of life, or social stigmatization (Mammen et al., 2024), other factors, such as physical limitations and how “bothersome” the symptom is by itself, may also play a role. In a cohort of 265 patients, those with less than 6 years since PD onset ranked their most troublesome symptoms in the descending order of most frequent complaints as slowness, tremor, stiffness, pain, and loss of the sense of smell (Politis et al., 2010). This contrasts with our study, where gait and balance problems represented one of the most common complaints, even during the early years of the disease; moreover, patients in our study did not rank the loss of the sense of taste and smell among their top complaints. In the study by Politis et al., the top 5 complaints in patients with ≥6 years of evolution were motor fluctuations (including dyskinesia), mood changes, drooling, sleep problems, and tremor (Politis et al., 2010). In our study, dyskinesia, speech difficulties, and sleep problems (insomnia) were more commonly reported in the top tiers for patients with more than 5 and 10 years of evolution, suggesting that these symptoms become particularly troublesome as the disease progresses. Dyskinesia and medication off states are also influenced by the effects of medications, as risk increases with disease progression and longer use of levodopa and dopamine agonists, making them more likely to be mentioned in the top tier after 5 years since symptom onset. However, tremor and gait problems remained as common complaints, even in patients with more than 10 years of disease progression. It is worth noting that we included drug-naïve patients and those already receiving treatment with levodopa and other antiparkinsonian drugs; however, this did not seem to influence the distribution of motor and non-motor complaints in our study.

In our study, we observed an overrepresentation of non-motor symptoms in the second tier for female patients. Biological differences between sexes may explain these differences. Previous studies have shown that female patients with PD appear to have a greater frequency of pain, fatigue, constipation, depression, anxiety, and insomnia, while male patients tend to have a greater frequency of sialorrhea, hypotension, urinary problems, sleep behavior disorders, and daytime sleepiness with faster progression of cognitive impairment (Georgiev et al., 2017; Cattaneo and Pagonabarraga, 2025). However, when considering patients with non-motor symptoms in the top tier, both male and female patients reported diverse motor and non-motor symptoms in the top three tiers (see Table 4).

Tremor was the most common troublesome symptom reported in our cohort. Few studies have emphasized that tremor is a prominent symptom in the early stages of PD with an impact on social interactions and the capacity to carry out activities of daily living and other tasks (Port et al., 2021; Heusinkveld et al., 2018). Tremor and other motor symptoms, such as gait problems, dyskinesia, or speech difficulties, may represent an important stigma (understood as bodily signs that expose something unusual or bad) for patients with PD, leading to feelings of shame and embarrassment due to undesirable self-image, loss of autonomy, and self-efficacy (Maffoni et al., 2017).

Non-motor symptoms are usually underrecognized and untreated in patients with PD. An online survey of 790 individuals with PD in the United Kingdom reported 2,295 issues related to this disease, where 1,358 (59.1%) were classified as motor, 859 (37.4%) as non-motor, and 78 (3.4%) as drug-related problems (Port et al., 2021). Non-motor symptoms also appear in the early years of the disease. One study showed that non-motor symptoms affect patients with early PD more commonly than controls (Baig et al., 2015). Non-motor symptoms may affect male and female patients differently, with problems in sexual function and difficulties with taste and smell more commonly reported by male patients (Picillo et al., 2013). Despite the short evolution time, these non-motor symptoms usually have an impact on quality of life and seem more common in patients with the PIGD phenotype than in those with the tremor-dominant phenotype (Baig et al., 2015). In our cluster analysis of patients with at least one non-motor complaint in their top three tiers, patients in the PIGD group commonly complained of fatigue, insomnia, and pain, whereas patients in the dyskinetic group had more commonly pain, anxiety, and insomnia as the most prominent non-motor complaints (Table 4).

One Japanese study enrolling 1,021 patients with PD and motor fluctuations showed that constipation was the most common non-motor symptom (85.4%), followed by sleep problems (73.7%), pain (72.7%), and daytime sleepiness (72%) (Maeda et al., 2017). In the present study, pain, anxiety, insomnia, fatigue, and altered mood were the most common non-motor symptoms, suggesting that this group of non-motor symptoms causes the greatest discomfort in patients with PD. While constipation or hyposmia are highly prevalent disorders in patients with PD, they were rarely mentioned as the most troublesome symptoms in our cohort.

Pain is a common but underrecognized complaint in patients with PD, causing a marked impact on quality of life (Cattaneo and Jost, 2023). Pain is reported to occur in approximately 60% of patients with PD (Young Blood et al., 2016). In our study, pain was the most common non-motor complaint in the top three tiers. This finding supports the notion that pain is one of the most disabling non-motor features in patients with PD and should be actively searched for at any stage of the disease. Urinary complaints are also common in patients with PD. They were the most common non-motor symptoms in a cohort of 117 patients with PD with a median evolution of 6 years (Tibar et al., 2018). While sexual dysfunction is reported in studies analyzing non-motor symptoms, this symptom was not spontaneously mentioned in our study, as shame or idiosyncratic concerns may limit patients from communicating this problem (Bronner and Vodušek, 2011).

Our study has limitations; first, we did not use a structured questionnaire for patients to rate each symptom to compare the burden level of each one. Instead, we asked patients to rate their most troublesome symptoms spontaneously, which we consider to be a reliable way to assess the burden of symptoms, as patients can easily and spontaneously name their top symptoms without hesitation. This may provide a strategic approach to treat such manifestations in an orderly manner, prioritizing the treatment of those manifestations causing greater concern or impairment. While this is not a validated patient-reported outcome measure, this strategy allows recognizing the burden of motor and non-motor symptoms in patients with PD and the potential contribution of neurological and psychiatric manifestations in each case, without neglecting all clinical aspects and comorbidities in patients with PD. This is a single-center study, which may limit the generalizability of the findings; however, patients from both sexes, diverse ages, and all types of PD were included, making the cohort representative. Moreover, as the cohort's mean disease duration was 6.61 years, early-stage PD may be underrepresented, a stage during which some prodromal non-motor symptoms, such as hyposmia or RBD, are prominent. Further studies should be conducted to assess the specific impact of each symptom on ADL, quality of life, stigma, etc., in a patient with PD. This will allow for a better understanding of why some symptoms confer a more troublesome character in patients with PD than others.

## Conclusion

Overall, tremor was acknowledged as the most troublesome symptom in patients with PD, but variability was high. Tremor, gait problems, and slowness were common top complaints at any time during the disease course. Speech difficulties, dyskinesia, and insomnia became prominent after 5 or 10 years of disease onset. Approximately 69% of patients had at least one non-motor symptom in their top-3 complaints, with pain being the most common complaint in this category. The presence of at least one non-motor symptom was predicted by an abnormal cognitive status, but not by sex, age, evolution time, or drug status. Among patients with at least one non-motor symptom, those with PIGD and dyskinesia had a higher frequency of fatigue, insomnia, pain, and anxiety, suggesting a clustering effect of non-motor symptoms with these motor presentations.

## Data Availability

The raw data supporting the conclusions of this article will be made available by the authors, without undue reservation.
