# Supplementary material for: Top motor and non-motor complaints in patients with Parkinson's disease
Source: Front Aging Neurosci. 2025 Oct 22;17:1664934. doi: 10.3389/fnagi.2025.1664934 (PMC12585944; doi:10.3389/fnagi.2025.1664934)
Supplement: Supplementary file 1 [file Data_Sheet_1.docx]

**Supplementary Table 1. Summary of frequency and hierarchy of “motor” symptoms in this cohort**

|  | 1st Tier  Responses, n=230 | 2nd Tier  Responses, n=229 | 3rd Tier  Responses, n=207 | Total  Responses |
| --- | --- | --- | --- | --- |
| Tremor | 80 | 15 | 28 | 123 |
| Gait problems | 37 | 53 | 25 | 115 |
| Slowness, low mobility | 16 | 19 | 10 | 35 |
| Gait freezing | 8 | 12 | 4 | 24 |
| Dyskinesia | 17 | 5 | 2 | 24 |
| Speech difficulties | 3 | 10 | 8 | 21 |
| Muscle weakness | 8 | 5 | 6 | 19 |
| Rigidity | 5 | 6 | 8 | 19 |
| Motor fluctuations | 6 | 3 | 1 | 10 |
| Dysphagia/drooling | 3 | 3 | 3 | 9 |
| Decreased dexterity | 2 | 2 | 4 | 8 |
| Writing difficulties | 1 | 5 | 1 | 7 |
| Postural abnormalities | 1 | 2 | 4 | 7 |
| Dystonia | 2 | 4 | 1 | 7 |
| Falls | 0 | 3 | 2 | 5 |
| Retropulsion | 0 | 1 | 0 | 1 |

**Supplementary Table 2. Summary of frequency and hierarchy of “non-motor” symptoms in this cohort**

|  | 1st Tier  Responses,  n=230 | 2nd Tier  Responses,  n=229 | 3rd Tier  Responses,  n=207 | Total  Responses |
| --- | --- | --- | --- | --- |
| Pain | 8 | 15 | 6 | 29 |
| Anxiety | 3 | 7 | 15 | 25 |
| Insomnia | 3 | 5 | 17 | 25 |
| Fatigue | 6 | 11 | 5 | 22 |
| Altered mood | 1 | 10 | 11 | 22 |
| Urinary problems | 0 | 9 | 6 | 15 |
| Cognitive complaints | 1 | 5 | 8 | 14 |
| Hypotension symptoms | 4 | 3 | 4 | 11 |
| Daytime somnolence | 4 | 4 | 3 | 11 |
| Paresthesia | 1 | 0 | 7 | 8 |
| Hallucinations, psychosis | 3 | 2 | 2 | 7 |
| Constipation | 1 | 3 | 3 | 7 |
| RBD | 0 | 1 | 3 | 4 |
| ICD | 2 | 0 | 1 | 3 |
| Apathy | 0 | 1 | 1 | 2 |
| Diaphoresis | 0 | 0 | 2 | 2 |
| Hyposmia | 0 | 1 | 0 | 1 |

ICD: Impulse control disorder; RBD: REM-sleep behavior disorder.

**Supplementary Table 3. Summary of frequency and hierarchy of “other” symptoms in this cohort**

|  | 1st Tier  Responses,  n=230 | 2nd Tier  Responses,  n=229 | 3rd Tier  Responses,  n=207 | Total  Responses |
| --- | --- | --- | --- | --- |
| Headache or migraine | 1 | 1 | 3 | 5 |
| RLS | 2 | 0 | 0 | 2 |
| Xerostomia | 0 | 1 | 1 | 2 |
| Hiccups | 1 | 0 | 0 | 1 |
| Myoclonus | 0 | 1 | 0 | 1 |
| Vertigo | 0 | 1 | 0 | 1 |
| Radiculopathy | 0 | 0 | 1 | 1 |
| Hyporexia | 0 | 0 | 1 | 1 |

RLS: Restless legs syndrome.
